# Babassu aqueous extract (BAE) as an adjuvant for T helper (Th)1-dependent immune responses in mice of a Th2 immune response-prone strain

**DOI:** 10.1186/1471-2172-12-13

**Published:** 2011-01-29

**Authors:** Rosane NM Guerra, Virgínia MG Silva, Luciana S Aragão-França, Pablo R Oliveira, Rodrigo Feitosa, Flavia RF Nascimento, Lain C Pontes-de-Carvalho

**Affiliations:** 1 Universidade Federal do Maranhão, Laboratory of Immunophysiology, São Luís, Brazil; 2 Centro de Pesquisa Gonçalo Moniz, Fundação Oswaldo Cruz, Salvador, Brazil

## Abstract

**Background:**

The aqueous extract of a Brazilian palm-tree fruit - the babassu - (BAE) exerts a clear immunostimulative activity *in vivo*. In the present work, the possibility that BAE can promote Th1 immune responses in mice of a Th2 immune response-prone strain - the BALB/c was investigated. BAE itself, and preparations consisting of *Leishmania amazonensis *promastigote extract (LE), adsorbed or not to Al(OH)_3_, and in the presence or not of BAE, were used as immunogens. LE and Al(OH)_3 _have been shown to preferentially elicit Th2 immune responses.

**Results:**

The addition of BAE to LE-containing immunogenic preparations, adsorbed or not to Al(OH)_3_, clearly promoted the *in vitro *production of interferon γ (IFN-γ), a major Th1-dependent cytokine, and not of interleukin (IL-)4 (a Th2-dependent cytokine), by LE-stimulated splenocytes of immunized BALB/c mice. It also promoted the *in vivo *formation of IgG2a anti-LE antibodies. However, immunization with LE by itself led to an increased production of IL-4 by LE-stimulated splenocytes, and this production, albeit not enhanced, was not reduced by the addition of BAE to the immunogen. On the other hand, the IL-4 production by LE-stimulated splenocytes was significantly lower in mice immunized with a preparation containing Al(OH)_3_-adsorbed LE and BAE than in mice immunized with the control preparation of Al(OH)_3_-adsorbed LE without BAE. Moreover, an increased production of IFN-γ, and not of IL-4, was observed in the culture supernatants of splenocytes, from BAE-immunized mice, which were *in vitro *stimulated with BAE or which received no specific *in vitro *stimulus. No differences in IL-10 (an immunoregulatory cytokine) levels in the supernatants of splenocytes from mice that were injected with BAE, in relation to splenocytes from control mice, were observed. The spontaneous *ex vivo *production of NO by splenocytes of mice that had been injected with BAE was significantly higher than the production of NO by splenocytes of control mice.

**Conclusions:**

Based on the results described above, BAE, or biologically active molecules purified from it, should be further investigated as a possible adjuvant, in association or not with aluminium compounds, for the preferential induction of Th1-dependent immune responses against different antigens in distinct murine strains and animal species.

## Background

Th1-dependent immune responses have been shown to protect mammals against many clinically relevant intracellular pathogens, such as *Mycobaterium tuberculosis*, *Leishmania *sp., and human herpes virus, and even against some extracellular pathogens, such as *Candida albicans *[1-4]. Adjuvants for Th1 immune responses could be, therefore, crucial components of a vaccine against this type of pathogens [5,6].

Different compounds have been tested for safety and adjuvant activity in human beings. Aluminum hydroxide, aluminum phosphate and Montanide^®^, a new generation of water in oil emulsions, are still the only adjuvants licensed for use in humans [7-9]. Lack of detailed data on safety, dose and routes of immunization has restricted to experimental animals the use of adjuvants that have been shown to be more potent or more directed to Th1 immune responses than the aluminum compounds [10-12].

The babassu (*Orbignya phalerata*, Mart, Arecaceae) is a palm tree commonly found in the northeast of Brazil. Babassu fruit flower is frequently used as food or folk medicine. Several important biological properties have been assigned to the babassu mesocarp flour, including an immunomodulatory activity [13]. It can increase the production of anti-insulin IgM antibodies in mice [14,15] and anti-thyroglobulin antibodies in rats [16]. In addition, the oral administration of an aqueous extract of babassu mesocarp to mice increased the production of inflammatory mediators (such as hydrogen peroxide, nitric oxide and TNF-α) by peritoneal macrophages *ex vivo *and the influx of leukocytes to the peritoneal cavity [17]. These known immunostimulative effects of the babassu aqueous extract (BAE) have motivated the present investigation on its possible immunoadjuvant activity. Immunization protocols utilizing immunogenic preparations that were likely to induce polarized Th2-dependent immune responses, namely *Leishmania *promastigote extract (LE) and Al(OH)_3 _gel-adsorbed LE [18-20], were used. As described below, the injection of BAE preferentially stimulated the Th1 component of the anti-LE and the anti-BAE immune responses in the immunized mice.

## Results

### Anti-LE antibody levels

**Figure 1:**
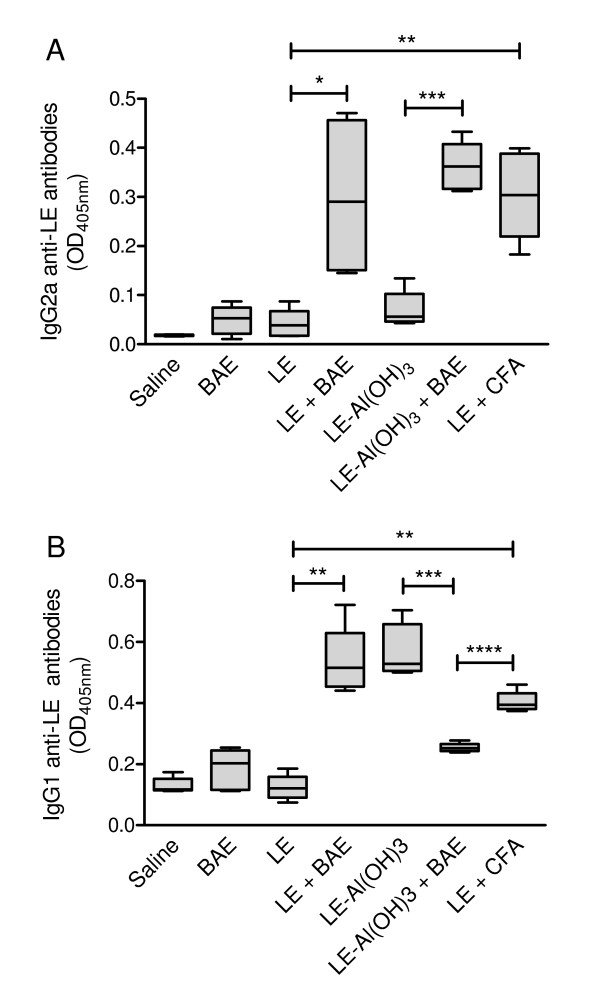
**IgG1 and IgG2a anti-*Leishmania *promastigote extract (LE) antibody levels in mice immunized with LE, associated or not with babassu aqueous extract (BAE)**. IgG2a (**A**) and IgG1 (**B**) antibodies were detected by ELISA as detailed in the Material and Methods, in sera prepared from blood samples collected 10 days after the end of the immunization. Groups of five to eight BALB/c mice were immunized by two subcutaneous injections of LE, associated with BAE **(LE + BAE**), or of Al(OH)_3_-adsorbed LE, also associated with BAE (**LE-Al(OH)**_**3 **_**+ BAE**), 14 days apart. Groups of control mice were also immunized with LE emulsified in complete Freund's adjuvant (**LE + CFA**), with LE without BAE (**LE**), and with Al(OH)_3_-adsorbed LE without BAE (**LE-Al(OH)**_**3**_), following the same immunization protocol. Each injection contained a total of 250 μg of LE and/or 5 mg of BAE. A negative control group was injected with a saline solution in place of the immunizing injections (**Saline**). The boxes represent the 10-90 percentile intervals and the median values of the results, and the vertical bars the value ranges. *P *< 0.001, Kruskal-Wallis test. *, *P *≤ 0.007; **, *P *≤ 0.003; ***, *P *≤ 0.009; ****, *P *≤ 0.016; Mann-Whitney U test.

The addition of BAE to the antigenic preparations (both to LE or Al(OH)_3 _gel-adsorbed LE) led to significantly increased levels of serum anti-LE IgG2a antibodies in the immunized mice (Figure 1A, groups "LE + BAE" and "LE-Al(OH)_3 _+ BAE") in relation to the corresponding control mice that did not receive BAE (Figure 1A, groups "LE" and "LE-Al(OH)_3_"). The co-administration of BAE also led to a statistically significant increase in anti-LE IgG1 antibodies in the LE-immunized mice that have not received Al(OH)_3 _(Figure 1B, group "LE + BAE") in relation to the mice that were immunized only with LE, without adjuvants (Figure 1B, group "LE"). However, the addition of BAE to the Al(OH)_3_-adsorbed LE preparation led to a statistically significant decrease in IgG1 anti-LE antibody levels in the immunized mice (Figure 1B, group "LE-Al(OH)_3 _+ BAE"), in relation to the mice that were immunized with Al(OH)_3_-adsorbed LE without BAE (Figure 1B, group "LE-Al(OH)_3_"). The results obtained with the use of BAE as adjuvant did not differ significantly from the results obtained with the use of CFA as adjuvant, both for IgG2a or IgG1 antibody levels (Figure 1). However, mice immunized with LE associated with both BAE and Al(OH)_3 _(Figure 1B, group "LE-Al(OH)_3 _+ BAE") had significantly lower levels of anti-LE IgG1 antibodies than mice immunized with CFA-emulsified LE (Figure 1B, group "LE + CFA").

### Cytokine responses in non-stimulated and in LE- and BAE-stimulated cultures

**Figure 2:**
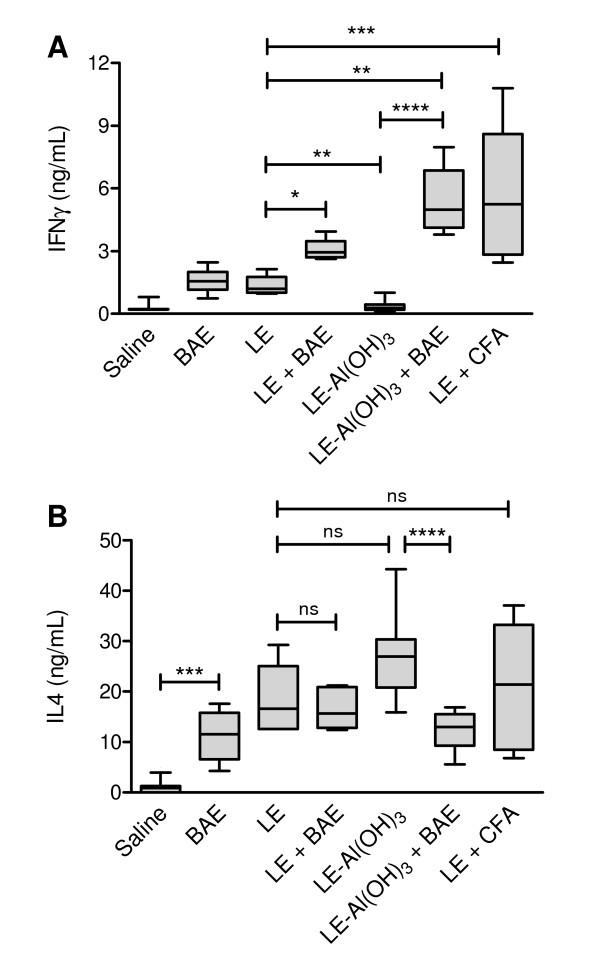
**Concentrations of IFNγ and IL-4 in the supernatants of splenocyte cultures from BALB/c mice immunized with *Leishmania *promastigote extract (LE), associated or not with babassu aqueous extract (BAE)**. Splenocytes (from spleens collected ten days after the end of the immunization) were stimulated with LE and concentrations of IFNγ (**A**) and IL-4 (**B**) in the supernatants were measured by ELISA. Groups of five to 12 animals were immunized by two subcutaneous injections of LE, associated with BAE (**LE + BAE**) or of Al(OH)_3 _gel-adsorbed LE, also associated with BAE (**LE-Al(OH)**_**3 **_**+ BAE**), 14 days apart. Groups of control mice were also immunized with LE emulsified in complete Freund's adjuvant (**LE + CFA**), with LE without BAE (**LE**), and with Al(OH)_3_-adsorbed LE without BAE (**LE-Al(OH)**_**3**_), following the same immunization protocol. Each injection contained a total of 250 μg of LE and/or 5 mg of BAE. A negative control group was injected with a saline solution in place of the immunizing injections (**Saline**). The boxes represent the 10-90 percentile intervals and the median values of the results, and the vertical bars the value ranges. *P *< 0.001, Kruskal-Wallis test. *, *P *≤ 0.004; **, *P *≤ 0.003; ***, *P *≤ 0.002; ****, *P *≤ 0.001; ns, non significant; Mann-Whitney U test.

When stimulated *in vitro *with LE, the splenocytes from the mice that were immunized with LE and received BAE as adjuvant (Figure 2A group "LE + BAE") produced significantly higher concentrations of interferon γ (IFN-γ) than the splenocytes from the mice that were immunized with LE without BAE (Figure 2A, group "LE"). The same result was observed when the mice were immunized with Al(OH)_3_-adsorbed LE and received BAE as adjuvant (Figure 2A, group "LE-Al(OH)_3 _+ BAE"). When stimulated *in vitro *with LE, the splenocytes from these mice produced significantly higher concentrations of IFN-γ than the splenocytes from mice that were immunized with Al(OH_)3 _gel-adsorbed LE without BAE (Figure 2A, group "LE-Al(OH)_3_"). The amounts of IFN-γ produced by splenocytes of the mice that received BAE, i.e., the mice that were immunized either with LE in the presence of BAE (Figure 2A, group "LE + BAE") or with Al(OH)_3_-adsorbed LE also in the presence of BAE (Figure 2A, group "LE-Al(OH)_3 _+ BAE"), were comparable to those produced by splenocytes from the mice that were immunized with CFA-emulsified LE (Figure 2A, group "LE + CFA"; *P *> 0.05, Mann-Whitney U test).

The production of interleukin (IL-)4 by LE-stimulated splenocytes was either not altered by the addition of BAE to the immunogenic preparations (in the case of mice immunized with LE; Figure 2B, group "LE + BAE", *P *> 0.05, Mann-Whitney U test), or was significantly reduced (in the case of mice immunized with Al(OH)_3 _gel-adsorbed LE; Figure 2B, group "LE-Al(OH)_3 _+ BAE", *P *< 0.001, Mann-Whitney U test), in relation to the corresponding control mice that did not receive BAE (Figure 2B, groups "LE" and "LE-Al(OH)_3_", respectively).

**Figure 3:**
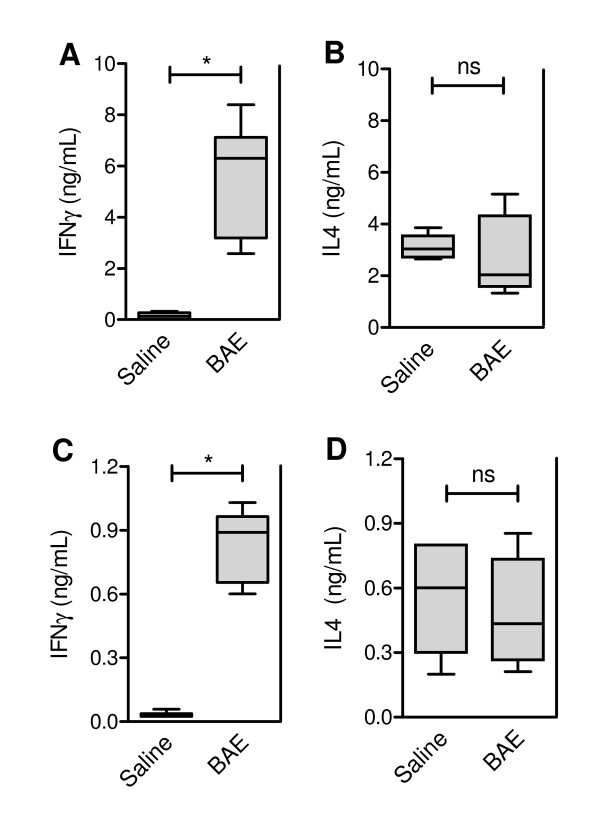
**Concentrations of IFNγ and IL-4 in the supernatants of splenocyte cultures from babassu aqueous extract (BAE)-immunized BALB/c mice**. Splenocytes (prepared from spleens collected ten days after the end of the immunization) were stimulated with BAE (**A **and **B**), or unstimulated (**C **and **D**), and the concentrations of IFNγ (**A **and **C**) and IL-4 (**B **and **D**) in the supernatants were measured by ELISA. Groups of five or six animals were immunized by two subcutaneous injections of 250 μg of BAE, 14 days apart (**BAE**). Negative control groups of five or six animals were injected with a saline solution in place of the immunizing injections (**Saline**). The boxes represent the 10-90 percentile intervals and the median values of the results, and the vertical bars the value ranges. *P *< 0.001, Kruskal-Wallis test. *, *P *≤ 0.004; ns, non significant; Mann-Whitney U test.

The production of IFN-γ, and not of IL-4, was significantly higher in BAE-stimulated cultures of splenocytes from BAE-immunized mice, than in splenocyte cultures from saline-treated control mice (Figure 3A and 3B). Spleen cells from the mice that had been immunized with BAE spontaneously produced, *ex vivo*, more IFN-γ than control saline-treated mice (Figure 3C). No statistically significant difference in the spontaneous production of IL-4 by splenocytes of BAE-immunized and saline-treated mice was observed (Figure 3D).

No statistically significant differences in IL-10 production by unstimulated, LE-stimulated, or BAE-stimulated splenocytes from the immunized and the non-immunized mice were observed (data not shown).

### Nitric oxide production

**Figure 4:**
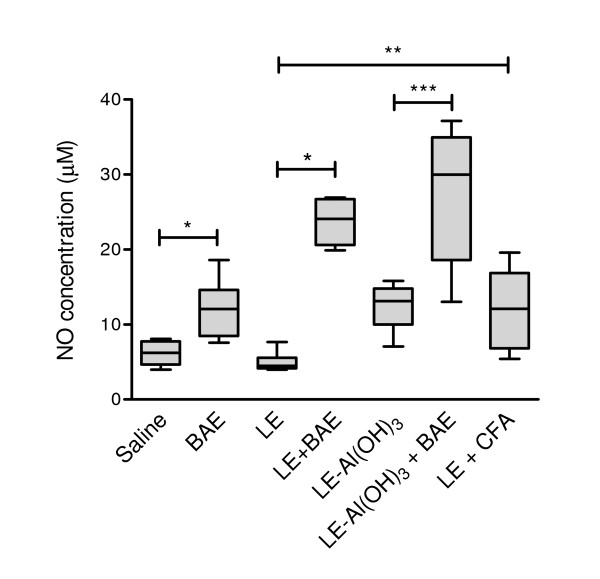
**Nitric oxide concentration in splenocyte cultures from mice immunized with *Leishmania *promastigote extract (LE) and babassu aqueous extract (BAE)**. Supernatants were obtained from unstimulated spleen cells, i.e, spleen cells that were not incubated with antigen *in vitro*. Spleens were collected ten days after the end of the immunization from groups of five to nine BALB/c mice that had been immunized with two subcutaneous injections of LE associated with BAE (**LE + BAE**) or of Al(OH)_3_-adsorbed LE associated with BAE **(LE-Al(OH)_3 _+ BAE)**, 14 days apart. Groups of control mice were also immunized with LE emulsified in complete Freund's adjuvant (**LE + CFA**), with LE without BAE (**LE**), and with Al(OH)_3_-adsorbed LE without BAE (**LE-Al(OH)_3_**), following the same immunization protocol. Each injection contained a total of 250 μg of LE and/or 5 mg of BAE. A negative control group was injected with a saline solution in place of the immunizing injections (**Saline**). The boxes represent the 10-90 percentile intervals and the median values of the results, and the vertical bars the value ranges. *P *< 0.001, Kruskal-Wallis test. *, *P *≤ 0.003; **, *P *≤ 0.005; ***, *P *≤ 0.016; Mann-Whitney U test.

The concentrations of nitric oxide in splenic cell supernatants from mice of the groups immunized with BAE or with BAE-containing preparations (Figure 4; groups "BAE", "LE + BAE", and LE-Al(OH)_3 _+ BAE) were significantly higher than in those from mice of the corresponding control groups, which were treated with saline only or immunized with preparations without BAE (Figure 4; groups "Saline", "LE", and LE-Al(OH)_3_, respectively). The concentrations of nitric oxide in splenic cell supernatants from the positive control mice, which were immunized with CFA-emulsified LE (Figure 4; group "LE + CFA"), were also higher than the nitric oxide concentrations in splenic cell supernatants from mice of their corresponding control group (Figure 4; group "LE").

## Discussion

The purpose of this study was to investigate the efficacy, as immunological adjuvant, of an aqueous extract of the babassu mesocarp. This adjuvant activity was investigated with the BAE in two different formulations: in soluble form or adsorbed to Al(OH)_3_. In both cases, its injection in mice stimulated a Th1-dependent immune response, both to itself or to LE, as demonstrated by: (i) a marked increase in circulating anti-LE IgG2a antibodies; (ii) an increased *in vitro *production of IFN-γ by LE-or BAE-stimulated splenocytes; (iii) an increased *ex vivo *production of IFN-γ by unstimulated splenocytes, and (iv) an increased *ex vivo *production of nitric oxide by splenic cells. As expected, the immunization of BALB/c mice with LE alone led to the significant production of IL-4 by LE-stimulated splenocytes [18]. The addition of BAE to the LE did not significantly augment this IL-4 production. This finding, therefore, does not support the possibility that, in addition to promoting Th1 immune responses, the BAE also enhances Th2 immune responses. On the other hand, a reduction in IL-4 production by the antigen-stimulated splenocytes, which could be expected from a Th1-polarising adjuvant, was not seen in these mice. However, additional results indicate that the BAE does preferentially enhance Th1 immune responses, and even reduces a Th2-dependent immune response. First, the addition of BAE to the immunogenic preparation significantly reduced the production of IL-4 by splenocytes from mice immunized with Al(OH)_3_-adsorbed LE. The reason why it would exert this inhibitory effect on the IL-4 production only when the anti-LE immune response is concomitantly stimulated by Al(OH)_3 _is not clear. It would not be surprising, however, that the LE and the Al(OH)_3 _would promote Th2 immune responses through different mechanisms, and it is possible that the Al(OH)_3_-induced mechanism could be more easily inhibited by immune manipulation than the LE-induced mechanism. Second, in mice injected with BAE alone, a recall immune response to antigens that were present in the BAE was observed. This response was characterized by significantly increased production of IFN-γ, and no significant differences in IL-4 production, by the mouse splenocytes when they were subjected to stimulation with BAE *in vitro*, in relation to the splenocytes of saline-injected control mice. An IFN-γ response was already significantly taking place *in vivo*, since splenocytes from the BAE-immunized mice spontaneously produced significant amounts of IFN-γ, and not of IL-4, *ex vivo*, in relation to splenocytes from control, saline-treated mice.

No differences in IL-10 levels in the supernatants of splenocytes from mice that were injected with BAE, in relation to splenocytes from control mice, were observed. This finding, which indicates that BAE is not inducing leukocytes to produce IL-10, could perhaps contribute to the stimulative effect of BAE on IFN-γ-producing immune responses, since IL-10 would tend to down regulate those immune responses [21].

The addition of BAE to the LE preparation also led to an increased production of IgG1 antibodies, apparently conflicting with the absence of increased IL-4 production by *in vitro*-stimulated splenocytes. However, IgG1 antibodies in mice have been shown to constitute two different molecular sub-populations, one which can sensitize mast cells and is dependent on IL-4 for its production, and another that does not bind to mast cells and depends on the Th1-inducing IL-12 cytokine to be produced [22,23]. Thus, whereas the presence of IgG2a antibodies can be considered a marker of an IFN-γ - producing Th1 immune response in most mouse strains, including the BALB/c [24,25], the implication of the presence of IgG1 antibodies, in terms of the nature of the underlying immune response, is much less clear.

In this study, the Th1 immune response-promoting activity of BAE was shown in mice of the BALB/c strain, which are Th2-biased [26]. It could be expected, therefore, that the Th1-promoting effect would be even more pronounced in other strains of mice.

Aluminium compounds are the most widely employed adjuvants for human beings [27]. Their effectiveness is associated with a deposit formation in the site of administration [28-30]. The mechanism responsible for the adsorption of antigenic proteins to aluminium adjuvants involves electrostatic forces and hydrophobic interactions, among other physicochemical phenomena [20]. However, the use of aluminium compounds as adjuvants is limited by their failure to stimulate cellular immune responses, including those mediated by cytotoxic T cells [19,20]. These compounds are known to induce a Th2 rather than Th1 immune responses [28-30], whereas CFA, not licensed for use in human beings, usually induces a mixed Th1-Th2 response [19,31]. These properties were confirmed in the present work.

The protective responses against some intracellular pathogens, such as *Leishmania *and *Mycobacterium*, are mediated by Th1 cells, both in mice [10,32] and in human beings [1,4]. There is, therefore, a need for highly efficacious, atoxic, Th1-promoting adjuvants, to be used in vaccine preparations against these and other pathogens. The Th1 cytokine profile induced by BAE qualifies it as a promising candidate to become one of these adjuvants. Indeed, the use of BAE as adjuvant led to increased *in vitro *production of IFN-γ and nitric oxide by splenocytes, and both these biologically active molecules have been shown to be positively involved in the intracellular killing of *Leishmania *and *Mycobacterium *[33,34]. In the case of *Leishmania*, however, the present results suggest that particular parasite antigens that do not induce Th2 immune responses, either by their intrinsic nature or by amino-acid sequence manipulation, should be used.

Advantages of the BAE, when compared with other adjuvants, include the ample availability of the babassu mesocarp, and its low cost of manufacture and ease of formulation. The data presented herein justifies further studies to identify the biologically active molecule(s) in the BAE. Since Th1 cytokines may inhibit the development of Th2 immune responses [32], the induction of Th1 immune responses by BAE may therefore also be useful in the control of diseases that are mediated by Th2 immune responses, such as allergic asthma and rhinitis.

## Conclusions

Based on the present findings, it was possible to conclude that: (i) BAE promotes preferentially a Th1-dependent immune response in a Th2-prone strain of mice; (ii) BAE, and/or biologically active molecules derived from it, deserve to be further investigated as possible adjuvants in candidate vaccines for leishmaniases and other diseases caused by intracellular pathogens, and possibly in the immunotherapy of diseases that are mediated by Th2 immune responses, such as allergic asthma and rhinitis.

## Methods

### Mice

Male, 8-12 week-old Balb/c mice, weighing 20-25 g, were from the Animal Breeding Unit of the Oswaldo Cruz Foundation, Salvador, Brazil. The animals were kept in ventilated cages, at 23 ± 1°C, with a relative humidity of 44-56%, and light and dark cycles of 12 h. They had free access to sterilized food and water. All procedures involving the animals were reviewed and approved by the institutional Ethics Committee in Experimental Animal Use, in accordance with the Brazilian College of Animal Experimentation.

### Babassu and *Leishmania *extracts

Babassu mesocarp flour was purchased from ASSEMA (São Luís, Maranhão, Brazil). This product was subjected to analysis of authenticity, integrity and purity, by physicochemical tests, including standard chromatographic techniques. Similarities in all physicochemical aspects were observed when it was compared with a mesocarp flour prepared in our laboratory [17] from babassu fruits collected from Pedreiras, State of Maranhão, Brazil (authenticated voucher specimen number 1135, filed in the Herbario Ático Seabra, São Luís, State of Maranhão, Brazil).

Eighty grams of the mesocarp flour were extracted during 1 h with 1 L of sterilized water, at 24°C, under constant stirring. The aqueous extract was filtered in 0.22-μm Millipore™ units and then diluted in 0.15 M phosphate-buffered saline, pH 7.2 (PBS), or culture medium, to the appropriate concentrations for the *in vivo *and *in vitro *studies. The composition of the extract, as measured by high-pressure liquid chromatography and gaseous chromatography, was: polysaccharides, 94.5%; proteins, 2.2%; lipids, 0.5%; monosaccharides and disaccharides, 1.8%; amino acids, < 1.0%.

Promastigotes from the MHOM/Br87/Ba125 *L. amazonensis *strain, derived from tissue amastigotes, were cultured at 23°C in Schneider's medium (Sigma-Aldrich Co., Saint Louis, MO, USA), pH 7.2, supplemented with 50 μg/mL of gentamycin and 10% of heat-inactivated fetal bovine serum (FBS; HIFCS, Gibco Laboratories, Grand Island, NY, USA). To prepare the LE, promastigotes were washed three times in ice-cold sterile saline, resuspended in ice-cold saline and lysed by exposition to ultrasound (10 × 1-min cycles on ice). The lysates were centrifuged at 16,000 g for 10 minutes at 4°C, the supernatants filtered on membranes with 0.22 μm-diameter pores (Millipore, São Paulo, Brazil) and immediately stored at -70°C in small aliquots. Their protein content was determined by Lowry's method [35].

### Immunizations

Al(OH)_3 _gel (Alhydrogel; Sigma-Aldrich Co., Poole, UK), at 1.3 mg/mL, was mixed with an equal volume of a solution containing LE (3.5 mg/mL) and BAE (80 mg/mL), incubated at room temperature for 20 minutes and vortexed before utilization. Groups of 5 to 9 animals were immunized by two subcutaneous injections of the Al(OH)_3 _gel-adsorbed extracts, separated by a two-week interval. Groups of 5-9 mice were also immunized, using the same protocol, with LE emulsified in complete Freund's adjuvant (CFA; 1 mg/mL of *Mycobacterium tuberculosis*, Sigma-Aldrich Co., Poole, UK), with LE mixed with BAE without Al(OH)_3 _gel, with Al(OH)_3 _gel-adsorbed LE without BAE, and with BAE. The amounts of LE and BAE in each injection were always 250 μg and 5 mg, respectively. A negative control group was injected with the diluent (saline) solution in place of the immunizing injections.

### Determination of antibody levels

Serum samples were prepared from blood collected ten days after the last antigen or saline injection, for determination of anti-LE IgG1 and IgG2a antibody levels by ELISA, as described previously [36]. Briefly, LE was diluted to a concentration of 40 μg/mL in carbonate-bicarbonate buffer (pH 9.6) and adsorbed onto wells of flat-bottomed ELISA strips (NUNC, Roskilde, Denmark) by overnight incubation at 4°C. The wells were then washed five times with PBS containing 0.05% of Tween 20 (PBS-T) and incubated with PBS-T containing 1% bovine serum albumin (PBS-TA) for 1 h at 37°C. Another wash as the described above was followed by the addition of a 1/100 dilution of mouse sera in PBS-TA. After 30 min at 37°C and further washing, 100 μL of peroxidase-conjugated goat anti-mouse IgG1 or IgG2a (PharMingen, San Diego, CA, USA), diluted 1:1000 in PBS-TA, were added to the wells and incubated for 1 h at 37°C. The wells were then washed five times with PBS-T and the colour was developed by adding 100 μL of a solution of tetramethylbenzidine and H_2_O_2 _and incubating in the dark for 20 min. The reaction was stopped with 2 N H_2_SO_4 _and the optical density (OD) for 450 nm wave-length light was measured.

### Cytokine quantification in spleen-cell cultures

Spleens were aseptically removed 10 days following the booster. Cell suspensions were prepared by gently teasing apart the splenic tissue in RPMI 1640 supplemented with 2 mM L-glutamine, 100 U/ml penicillin, 100 μg/mL streptomycin, 0.05 mM 2-mercaptoethanol, and 10% FBS (Life Technologies, Paisley, UK). Viable cells were enumerated in a Trypan blue exclusion assay. Cytokine production was assessed by culturing splenocytes (10^6^/mL) in 24-well flat-bottom tissue culture plates (Costar, Cambridge, MA, USA). All samples were incubated in duplicate (500 μL/well) with LE (40 μg/well) or BAE (50 μg/well) or with RPMI 1640 medium alone. Supernatants were harvested after 24 and 48 h incubation at 37°C and 5% CO_2_, and stored at -20°C until assayed. IFN-γ, IL-4, and IL-10 were quantified by commercial capture ELISA kits, according to the manufacturer's directions (eBiosciences, San Diego, CA, USA). The detection limits of the ELISAs were 2 pg/mL for IFN-γ and IL-4, and 4 pg/mL for IL-10. Cytokine concentrations in the cell culture supernatants were determined by interpolation of the obtained ODs (for 492 nm-wave length light) values in cytokine standard curves (which had *r *≥ 0.99).

### Nitric oxide concentration

Fifty-μL volumes of supernatants from unstimulated splenocytes, incubated for 24 hours *in vitro *at 37°C, 5% of CO_2 _atmosphere and cell density of 5.2 × 10^6^/mL, were collected and incubated with an equal volume of Griess reagent (1% sulfanilamide, 0.1% naphthalene diaminedihydrochloride, 2.5% H_3_PO_4_) for 10 min at room temperature, to allow the accumulation of nitrite (a stable end product of the NO reaction) [37]. The absorbance of 550 nm wave-length light was determined. Conversion of absorbance to μM of NO was done by interpolation into a standard curve obtained with known concentrations (5-60 μM) of sodium nitrite in RPMI medium.

### Statistical analysis

The statistical significances of differences among experimental groups were determined, with the use of the SPSS Statistics 17.0 program, by the Kruskal-Wallis and Mann-Whitney U tests for whole experimental results and pairwise comparisons, respectively. Values were considered statistically significant when *P *≤ 0.05.

## Authors' contributions

All authors carried out experimental procedures, interpreted the results and/or helped to draft the manuscript. All authors read and approved the final manuscript.
